# An Evaluation of a Dietitian‐Led Webinar for Adults With Coeliac Disease: A Positive Change in Gluten‐Free Food Knowledge and Behaviours

**DOI:** 10.1111/jhn.70350

**Published:** 2026-09-23

**Authors:** Leah Seamark, Olivia Radcliffe, Marieanne Williams, Jim Gotto, Bridie Watson, Jenna Trudgeon, Sue Reeves, Yvonne Jeanes

**Affiliations:** ^1^ Somerset NHS Foundation Trust Community Dietetics Bridgwater UK; ^2^ The Gut Clinic Somerset UK; ^3^ North Bristol NHS Trust Westbury‐On‐Trym UK; ^4^ University of Roehampton London UK; ^5^ British Dietetic Association, Birmingham UK; ^6^ Kings College London, Holborn UK

**Keywords:** coeliac, dietitian, digital, gluten

## Abstract

**Introduction:**

The only treatment for coeliac disease is the exclusion of all gluten from the diet. Adherence to a gluten free (GF) diet requires knowledge acquisition and changes in behaviours. The service evaluation aimed to evaluate the impact of a dietitian‐led webinar for adults with coeliac disease.

**Methods:**

The pre‐recorded dietitian‐led webinar had been developed by specialist dietitians and gastroenterologists and was integrated into a patient pathway. A pre‐ and post‐webinar online survey was designed to evaluate the impact of the webinar for adult patients with coeliac disease. Patients were invited to complete an online survey prior to accessing the webinar. Four weeks later, the patients were invited to complete the post‐webinar survey.

**Results:**

The webinar was accessed by 1050 adults with coeliac disease, of which 57% were diagnosed within the preceding 8 weeks. In patients who completed both surveys, there was a significant improvement in confidence (*p* < 0.001) and knowledge (*p* < 0.001) to follow a GF diet (*n* = 165). Among patients who reported gastrointestinal symptoms on gluten ingestion (*n* = 71), a greater proportion reported satisfactory symptom relief after watching the webinar (*p* = 0.02). Behaviours to minimise gluten ingestion were positively impacted, whereby a higher proportion of patients reported to *‘doing things to avoid eating gluten when preparing in the home’* and ‘*having GF foods on hand while they are out’* (*p* < 0.05).

**Conclusion:**

The dietitian‐led webinar enabled timely access to high‐quality resources from specialists. Access to the webinar positively impacted knowledge and behaviours to minimise gluten ingestion. This is a practical and inexpensive solution to enable specialist dietitians to utilise their skills and finite time for appointments with patients with more complex presentations.

## Practitioner Points

- Pre‐recorded dietitian‐led webinar provides immediate access to evidence‐based, high‐quality practical information for adults diagnosed with coeliac disease, from a specialist dietitian.
- Efficient use of specialist dietitian time; providing patients with an introduction to dietary management of coeliac disease with positive changes to self‐manage the GF diet.
- Practical and inexpensive solution that can help to reduce pressure on primary and secondary healthcare resources.

## 1 Introduction

Coeliac disease has a global prevalence of approximately 1.4% [1] and is characterised by a permanent loss of tolerance to dietary gluten resulting from an autoimmune response in genetically predisposed individuals. The clinical presentation of coeliac disease is highly variable and can include gastrointestinal symptoms and extraintestinal manifestations. With no cure, a lifelong, gluten‐free (GF) diet is the only treatment and, for the vast majority, results in complete clinical and histological recovery [2].

A GF diet excludes wheat, barley and rye, grains ubiquitously used in breads, pasta, cakes and pastries. Adherence to a GF diet is widely recognised as challenging, necessitating substantial knowledge acquisition and behavioural modification by individuals [3]. External barriers include a lack of widespread availability and high cost of GF foods, risk of cross‐contact and the inherently restrictive nature of the diet, collectively impacting quality of life and contributing to the high treatment burden people with coeliac disease report [3]. Dietetic intervention is comprehensive and aims to enable patients to adhere to a nutritionally adequate GF diet while minimising the overall dietary treatment burden [4].

Dietetic provision for adults with coeliac disease has evolved over time and substantially diversified [5, 6, 7]. In a 2019 UK survey of dietetic services, the majority of dietetic departments offered individual appointments for adults newly diagnosed with coeliac disease (60%), 33% offered group sessions and 5% offered online film/pre‐recorded webinar as their first point of contact with the dietetic service [7]. The study observed a substantial reduction in the number of patients being able to access dietetic services within 4 weeks of diagnosis compared with 12 years earlier; 22% versus 84% [7]. Patients newly diagnosed with coeliac disease need to self‐manage without specific guidance for several weeks until they can access a dietetic service.

A substantial volume of information related to GF living is available online, including on social media platforms; however, the quality of this content is largely unregulated and of varying quality. In 2024, a survey of 675 adults with coeliac disease reported that a third of respondents had experienced misinformation from the internet and social media related to coeliac disease or living GF [8]. There are clear benefits of the online coeliac communities; for example, people who regularly use social media, 64% found it to be very/extremely useful for providing information on new GF foods and 59% for travelling or eating out GF. A 2025 UK government reports the digital era is democratising knowledge, recognising that people have long turned to their phones as a first port of call for health information [9].

There is an increasing number of dietitians on social media platforms, and dietitians need to continue creating high‐quality digital content to support instantly accessible dietary information for people living GF. In 2018, Cardiff and Vale University Hospital incorporated a YouTube video for adults newly diagnosed with coeliac disease; 77% watched the video within five days of receiving their diagnosis, reporting that their knowledge of where gluten is found in the diet had improved [10]. In the US, dietitians have embraced TikTok and are successfully engaging children and adolescents with their coeliac disease educational content [11]. Muhammad et al used semi‐structured qualitative interviews to explore the potential challenges faced by adults with coeliac disease in relation to outpatient clinics and the acceptability of alternatives. Over half of participants considered an online film link was favoured in relation to acquiring knowledge, but not as a replacement for an individual healthcare professional appointment [12].

Very few studies have evaluated the impact of online interventions for people with coeliac disease. A couple of six‐week interventions have reported favourable outcomes; an Australian six‐module online course improved GF food knowledge and GF dietary adherence, though high levels of attrition were reported [13]. A six‐week intervention delivered by social media in Spain reported improvements in areas of knowledge related to adhering to a GF diet [14]. Outside the field of coeliac disease, the authors found three published evaluations of dietitian‐led webinars. People who watched a webinar for the dietary management of irritable bowel syndrome (IBS) indicated that access to accurate and reliable information, being able to rewatch the webinar and having instant access to specialist information were important to them [15]. A dietitian‐led pre‐recorded webinar to introduce the low FODMAP diet for adults with IBS reported a positive impact on patient symptoms [16], and a dietitian‐led webinar series for parents of children with fussy eating behaviours indicated that they reduced mealtime stress, increased food exposure and helped to foster a positive mealtime environment [17]. The ability for adults diagnosed with coeliac disease to have instant access to reliable dietary information could minimise the hazard of incorrect self‐education and assist dietary adherence.

This study aimed to evaluate a dietitian‐led pre‐recorded coeliac disease and the GF diet webinar, in terms of patients’ GF food knowledge, confidence and behaviours to promote GF dietary adherence.

## 2 Methods

A single‐group pre‐post study design was used to evaluate the effectiveness of a dietitian‐led pre‐recorded webinar focused on the GF diet for adults with coeliac disease. Individuals with a diagnosis of coeliac disease who accessed the webinar between March 2022 and November 2024 were invited to complete an online pre‐webinar survey prior to accessing the webinar.

### 2.1 Developing the Webinar

An initial webinar was developed in 2017 by specialist dietitians and gastroenterologists and evaluated for acceptability. A presentation was developed by specialist gastroenterology dietitians working in the Somerset NHS Foundation Trust, which was used as the visual aid, with conversational content between specialist dietitians and gastroenterologists. Content included information on what coeliac disease is, the diagnostic procedure, a GFD, food labelling, cross‐contact, tips for eating out and when on holiday. The content was edited and uploaded to www.patientwebinars.co.uk. The webinar was a 1‐hour‐long video and was included in the patient pathway. To optimise the benefit to others, the webinar was not branded, was free to access, and could be accessed outside of the UK.

Eighty‐three patients/relatives/carers/friends' responses were received for a short survey directly after watching the webinar (September 2018–March 2022). The majority (93%) reported the webinar was ‘very easy/easy’ to access, 93% reported they were ‘very likely/likely’ to recommend the webinar to someone who has been diagnosed with coeliac disease, and 96% reported they were ‘very satisfied/satisfied’ with the overall content of the webinar. The webinar was updated in 2021, along with the Somerset NHS Foundation Trust coeliac patient pathway. Following a diagnosis of coeliac disease, patients were referred to the local dietetic department. Patients were then sent a letter directing them to watch the webinar, and an appointment with a dietitian was offered, aiming for an appointment around 8 weeks from diagnosis. Patients who did not have access to the appropriate technology to watch the webinar were encouraged to contact the department, so an earlier appointment with a dietitian could be arranged. The project did not require ethical approval according to the national health research authority tool, and it was registered as a service evaluation in accordance with local quality and assurance compliance.

### 2.2 Survey Recruitment and Design

Participants may have been directed to the webinar as part of their local coeliac disease pathway, or directed there by other healthcare professionals aware of the resource or independently found the webinar via an online search engine. Individuals were asked if they were the patient/relative/carer/friend. Only patients were invited to complete the pre‐webinar survey. Patients with coeliac disease who declined to take part in the survey were still required to answer anonymous demographic questions (age, ethnicity, gender and time since diagnosis) before being able to access the webinar. The inclusion criteria were patients with a diagnosis of coeliac disease from a healthcare professional and 18 years of age or over.

Patients who consented to be included in the study were contacted 4‐weeks after they completed the pre‐webinar survey with a post‐webinar survey, sent via text and/or email. Reminder texts and emails were sent to participants one week later if they had not completed the survey. Patients’ pre‐ and post‐webinar responses were matched. Inclusion criteria for analysis required patients to have watched the full webinar and not to have visited a dietitian before completing the post‐webinar survey.

The pre‐ and post‐survey content were designed by specialist dietitians and informed by published studies and hosted via the survey platform Questback (Questback, Bridgeport, CT, USA). Questions assessing patient self‐reported confidence and behaviours were adapted from a published study and rated on a 5‐point Likert scale [18]. One question assessed patient confidence to follow a GF diet (1 = not at all confident, 2 = not confident, 3 = not sure, 4 = confident and 5 = very confident). The GF food knowledge questionnaire consisted of seventeen foods, modified to include foods recognised in the UK [19] (Supplementary Information S1); patients indicated if the food was ‘allowed’, ‘to question’ or ‘not allowed’. The score was cumulative and ranged from 0 to 17, with 17 being the most knowledgeable in relation to GF foods.

Survey to ascertain access to the webinar: In two hospital trusts, adults newly diagnosed with coeliac disease were also sent, along with the webinar information, a paper questionnaire to return in a stamped address envelope, to collect data on access to the webinar.

Statistical analysis: Categorical data pre‐ and post‐webinar were dichotomised, and McNemar's test was applied; GF food knowledge scores were tested for normality and values are presented as median (inter‐quartile range) with the Wilcoxon signed‐rank test to compare pre‐ and post‐scores. A *p‐*value of < 0.05 indicated a significant difference.

## 3 Results

**Figure 1:**
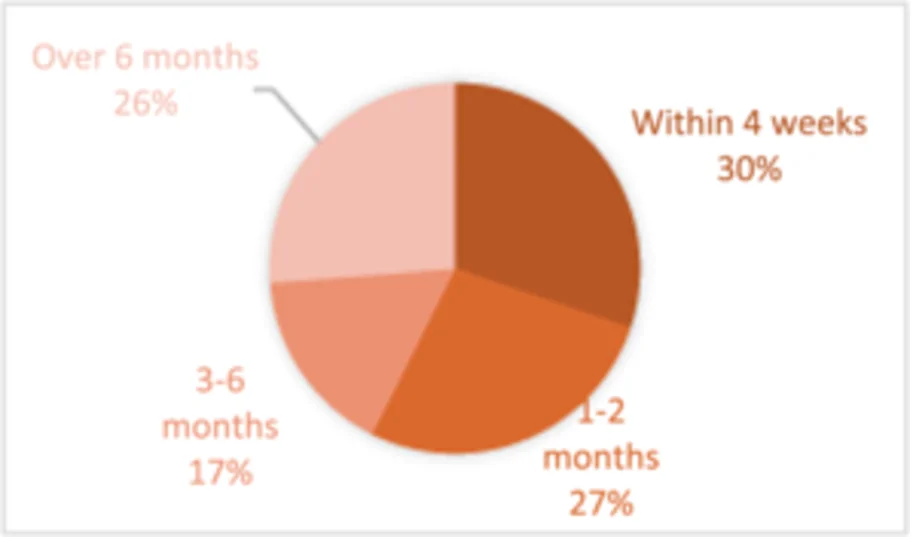
Time from diagnosis to watching the webinar (*n* = 1000).

The coeliac disease webinar was accessed 2013 times between March 2022 and November 2024; 211 people reported watching to help a family member, friend or someone they care for. The webinar was accessed by 1050 people with coeliac disease; 1000 responses had demographic data, which also included length of time since diagnosis. Over half of the people accessing the webinar (57%) had received their diagnosis within the previous 8 weeks, and 30% were within 4 weeks of diagnosis (Figure 1). The majority (87%) were planning to have an appointment with a dietitian in the near future.

**Figure 2:**
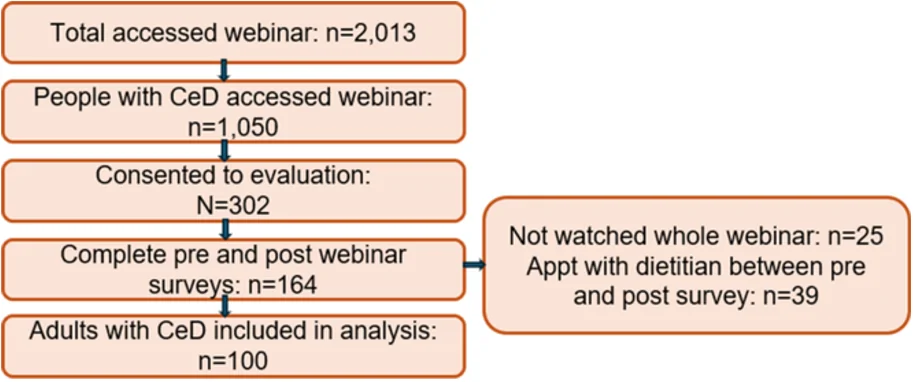
Number of people who accessed the webinar and completed the pre‐ and post‐webinar surveys.

Twenty nine percent of patients (302 respondents) consented to be part of the service evaluation and completed the pre‐webinar survey; of these, 164 also completed the post‐webinar survey (Figure 2). Of the 164, 139 patients watched the whole webinar (13 watched part, 12 did not watch the webinar), and a further 39 patients had attended an appointment with a dietitian between pre‐ and post‐survey dates. Thus, 100 patients were included in the pre‐ and post‐webinar comparison analysis. The average time between pre‐ and post‐survey completion was 35.6 ± 13.8 days.

The ‘access to the webinar’ paper survey received 42 responses: 14 patients had not watched and 5 partially watched the webinar, and the reasons for not watching the whole webinar were poor internet access (*n* = 4) and not enough time (*n* = 4).

### 3.1 Characteristics for Patients Who Completed the Pre‐ and Post‐Webinar Survey

Patients were predominantly white (95%) and female (71%); similar characteristics were observed in people who did not access the webinar (supplementary file S1). Over two‐thirds of participants had been diagnosed with coeliac disease within 8 weeks of watching the webinar (*n* = 71).

The majority of patients who completed the pre‐ and post‐webinar surveys reported symptoms when they ingested gluten (71%). There was a large variation in both symptoms reported and severity of symptoms, with flatulence and tiredness being the most reported to have a severe impact. Twenty one percent of all patients reported knowingly consuming gluten since diagnosis, and a further 26% had consumed gluten by accident. The majority of participants (82%) reported being ‘very concerned/concerned’ about the impact of consuming gluten on their health.

### 3.2 Positive Changes in Knowledge and Behaviours Post‐Webinar

**Figure 3:**
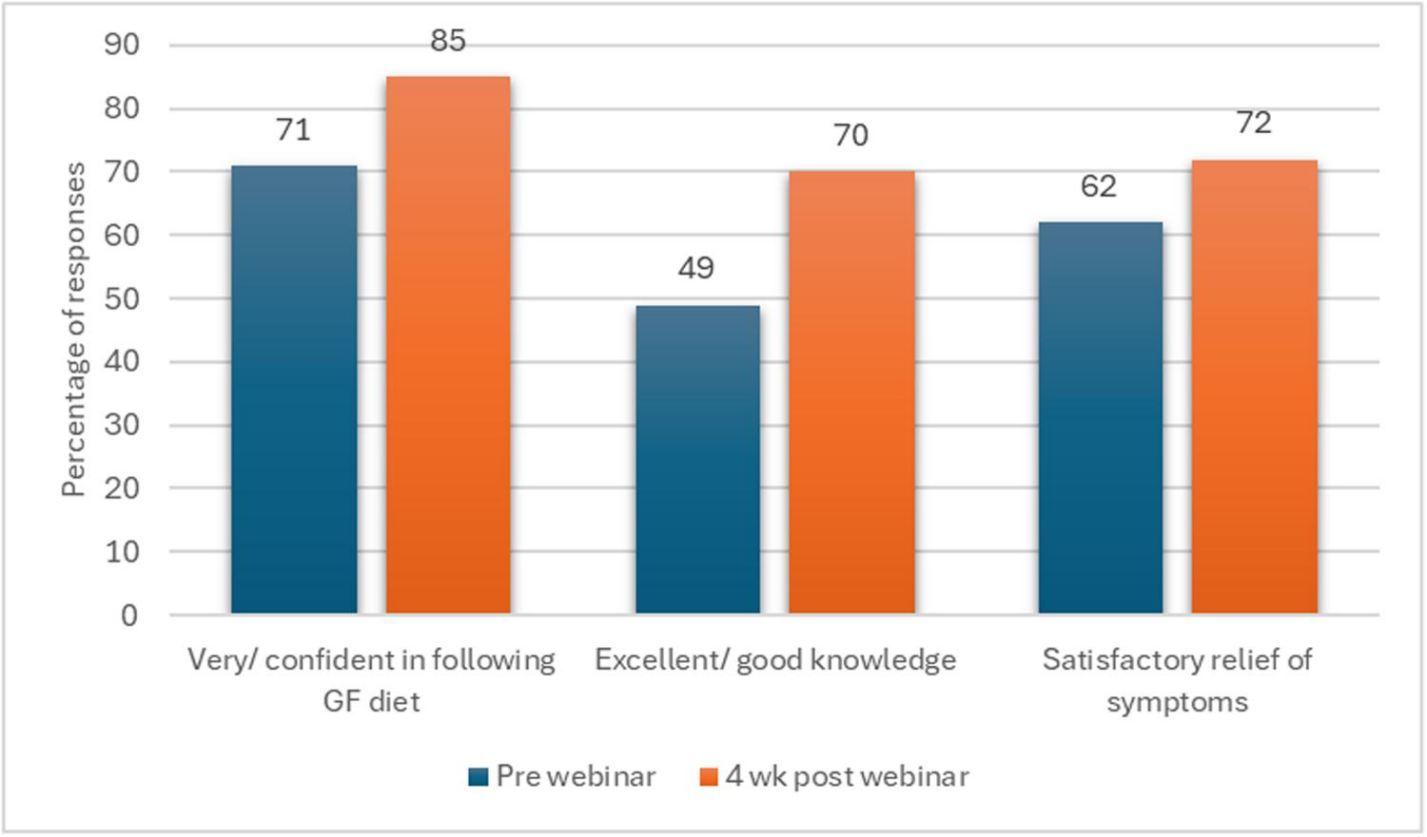
Improvements in symptoms, confidence and knowledge after watching the webinar. Significantly greater proportion reported to be ‘very confident/confident’ following GF diet (*p* = 0.001), to have ‘excellent/good knowledge’ of GF diet (*p* < 0.001): n = 100 and sub‐group analysis of *n* = 71 who reported symptoms on ingestion of gluten, a greater proportion reported satisfactory relief of gastrointestinal symptoms post‐webinar (*p* = 0.02).

Significant improvements were observed in patients' confidence and knowledge to follow a GF diet compared with pre‐webinar responses (Figure 3). In the sub‐set of patients who were diagnosed within 8 weeks preceding the webinar, there was a relatively larger increase in the proportion who were ‘very confident/confident’ (*n* = 71; from 48% to 62%; *p* = 0.001) and who reported ‘excellent/good knowledge’ to follow a GF diet (*n* = 71; from 29% to 50%; *p* < 0.001) after watching the webinar. In the subset of patients who reported gastrointestinal symptoms on ingestion of gluten, there was an improvement in the proportion reporting satisfactory relief of symptoms compared with pre‐webinar responses (Figure 3).

GF food knowledge scores significantly improved compared with pre‐webinar scores: median (IQR) score of 11(9–12) increased to 12(9–14); *p* < 0.001. In the subgroup of patients who were diagnosed within the preceding 8 weeks of watching the webinar, their GF food knowledge scores increased from 10 (8–11) to 11 (8–13); *p* < 0.001. Additionally, a small group of patients (*n* = 29) who reported they were ‘not sure/not at all confident’ in the GF diet had a relatively lower initial GF food knowledge score, with a significant improvement post‐webinar: from 8 (5–10) to 10 (8–13); *p* = 0.004.

Codex wheat starch, buckwheat and modified maize starch were GF foods that a significantly higher proportion of patients correctly identified compared with the pre‐webinar responses (codex wheat starch: from 5% to 24%; *p* < 0.001, buckwheat: from 25% to 39%; *p* = 0.02, modified maize starch: from 38% to 58%; *p* = 0.001). Spelt was the only gluten‐containing food that a higher proportion of patients correctly identified as containing gluten in the post‐webinar survey compared with the pre‐webinar responses (from 49% to 66%; *p* = 0.003).

**Figure 4:**
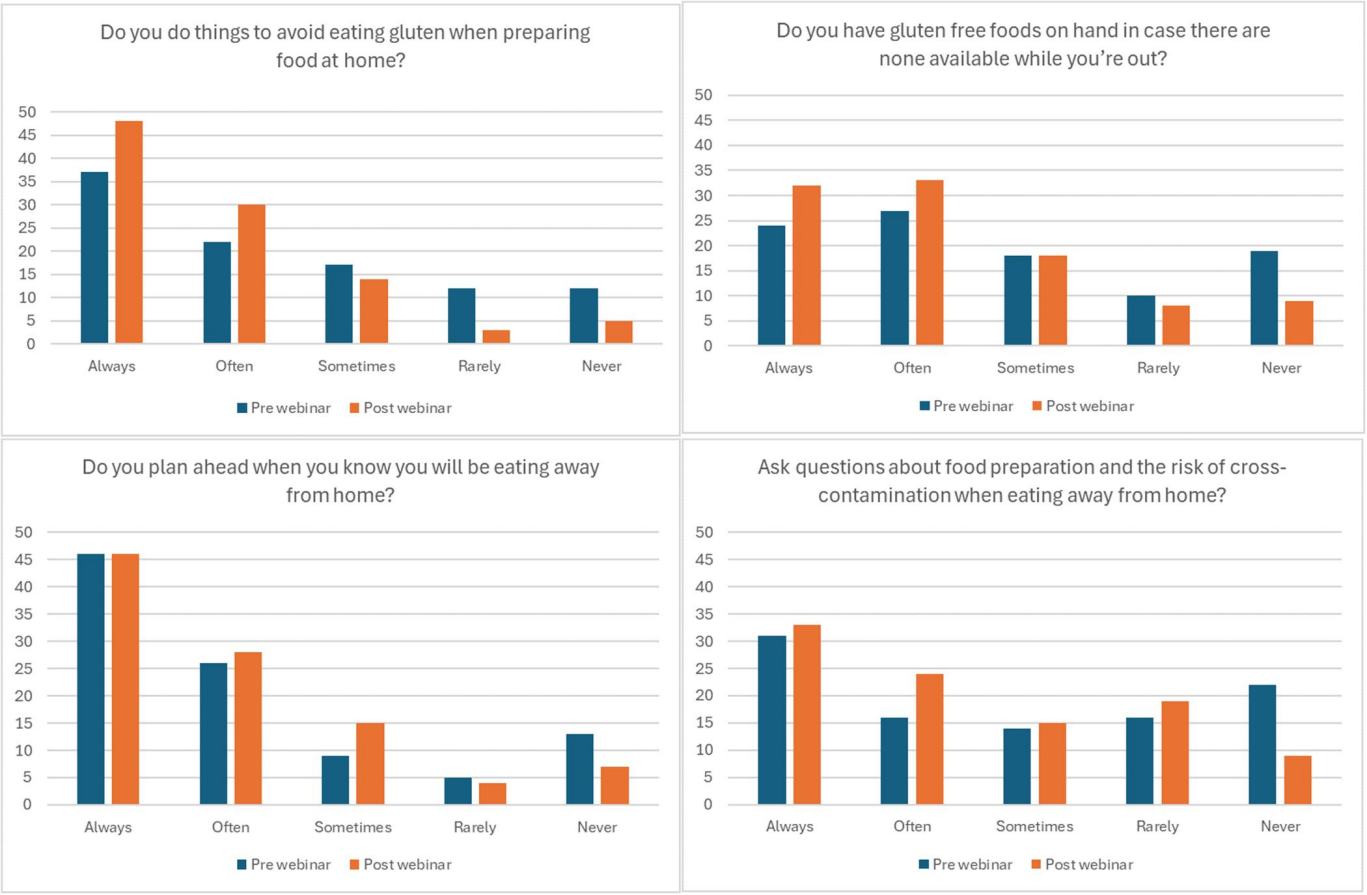
Number of patient responses to behaviours to promote GF diet adherence before and after watching the webinar.

There was a significant increase in patients reporting to ‘always/often’ do things ‘*to avoid eating gluten when preparing in the home’* and ‘*having GF foods on hand while they are out’* (Table 1). Likewise, there was a significant reduction in people reporting ‘rarely/never’ doing these behaviours after the webinar (Table 1, Figure 4). Most participants reported ‘always or often’ *‘read ingredients lists on food labels’* before watching the webinar (91%); this remained high after watching the webinar (96%). Whereas before watching the webinar, over a third of participants (38%) generally did not ‘*ask questions about food preparation and the risk of cross‐contamination when eating away from home’*, and after the webinar, there was a trend towards more participants engaging in this behaviour (Table 1), though not reaching significance (*p* = 0.089).

**Table 1:** Responses to behaviours which promote GF dietary adherence.

Do you:	% Often/Always		*p* value	% Rarely/never		*p* value
	Pre‐webinar	Post‐webinar		Pre‐webinar	Post‐webinar	
Read ingredient lists on food labels?	91	96	0.125	7	2	0.063
Read ‘may contain’ statements on food labels?	79	87	0.057	9	5	0.289
Do things to avoid eating gluten when preparing food at home?	59	78	< 0.001	24	8	< 0.001
Plan ahead when you know you will be eating away from home?	72	74	0.850	18	11	0.077
Tell people who are preparing food for you about your coeliac disease and need for a GF diet?	77	86	0.057	17	10	0.092
Ask questions about food preparation and the risk of cross‐contamination when eating away from home?	47	57	0.064	38	28	0.089
Have GF foods on hand in case there are none available while you're out?	51	64	0.026	29	17	0.004

*Note: N* = 100, analysis by the McNemar test.

For the subgroup of patients who were diagnosed within the preceding 8 weeks of watching the webinar (*n* = 71), significantly more indicated that they ‘often/always’ *do things to avoid eating gluten when preparing food at home’ p* = 0.03) compared to before the webinar.

## 4 Discussion

Adults with coeliac disease had increased GF food knowledge and engagement in behaviours to promote GF dietary adherence after watching a dietitian‐led pre‐recorded webinar. A large proportion of participants had a recent diagnosis of coeliac disease, with the webinar providing them with timely, evidence‐based information from a specialist dietitian near to the time of their diagnosis. The challenge of access to a specialist dietitian is acknowledged in the literature, with recognition that there is a lack of dietitians who have a specialist interest in coeliac disease [6]. Studies have shown specialist dietitians with expertise in coeliac disease positively impact patient outcomes, for example, through identifying involuntary gluten ingestion [20], improving nutritional adequacy and reducing the dietary burden [4]. The webinar enabled access to specialist dietetic intervention and helped empower patients to self‐manage their condition. Previous research highlighted that patients appreciate the ability to ‘rewatch the webinar’, which is an advantage of pre‐recorded webinars versus face‐to‐face or remote dietetic consultations [16].

A strength of the evaluation is the real‐world setting for data collection, demonstrating the intervention has a direct impact on patient outcomes. The development of a dietitian‐led webinar for patients aligns with the NHS England long‐term plan of digitising healthcare and reporting on outcomes from services/interventions [9]. An evaluation of a dietitian‐led webinar for IBS indicated that the costs of development and delivery of the webinar were more than offset by time savings, which were sufficiently significant to allow the expansion of the service to other patient groups [15]. Likewise, the coeliac pathway whereby patients access the webinar before a one‐to‐one appointment reduces the time required to provide general information on coeliac disease and GF foods, which is covered in the webinar. It does not replace individualised dietetic care. The webinar enables the subsequent dietetic appointment(s) to focus on individualised content, for example, culturally appropriate items, food insecurity concerns, comorbidities, as well as to explore the impact on quality of life and mental well‐being.

A lower income and education level have been linked to reduced internet access and limited use of eHealth technology, raising concerns that an increased emphasis on digital care could widen existing socioeconomic gaps in access [21]. In response to this, the NHS coeliac pathway incorporates triage within dietetic services so that adults with coeliac disease who have complex medical needs, learning disabilities, limited English proficiency, or no digital access can be offered timely one‐to‐one appointments. This approach ensures that the level of support for the first dietetic consultation is tailored to individual needs, helping to maintain an equitable service.

The dietitian‐led webinar offers freely accessible, high‐quality, evidence‐based guidance for living GF within the digital space—an area where patients frequently seek support, yet where healthcare‐led resources remain scarce [8]. Concerns about the reliability of online information are longstanding: McNally et al. reviewed coeliac disease content from 98 websites and found that many were insufficiently accurate, comprehensive, or transparent to be considered trustworthy for patients [22]. Similar issues are seen across social media, where personal narratives dominate; in one review of a video‐based platform, 47% of content consisted of personal stories, and only a single video was created by a healthcare professional [23].

The improvement in gastrointestinal symptoms observed four weeks after the webinar is clinically important and could be due to improved GF dietary adherence, though not measured directly in this service evaluation. GF dietary adherence is multifactorial and, with no gold standard, very difficult to assess. Our evaluation focused on measurable outcomes, such as GF food knowledge and behaviours related to dietary adherence. This is similar to the newer GF dietary adherence tools that have recently been published [4], for example, the inclusion of ‘label reading skills, which are necessary to successfully avoid gluten and can help to address accidental gluten exposure’. A high proportion of patients reported reading ingredients and ‘may contain’ on food labels pre‐ and post‐webinar. We did not assess the participants' ability to understand food labels, which has been associated with GF dietary adherence [24].

Both patients’ self‐reported GF food knowledge and GF food knowledge scores improved after watching the webinar. Similar to a study in Canada, the GF ingredient most mistaken to contain gluten was codex wheat starch [25]. It is important for patients to be aware of the GF foods to minimise over‐restriction and reduce the burden of the GF diet. The incorrect identification of gluten‐containing foods highlights the need for ongoing dietetic intervention and patient learning beyond watching the webinar. Insufficient knowledge to identify gluten in a product could subsequently lead to inadvertent gluten ingestion. There is a paucity of studies reporting outcomes from interventions to promote GF dietary knowledge and adherence. A telephone healthcare appointment has been shown to increase GF food knowledge scores [26], as has a six‐week online course reported improvements in a 14‐ingredient knowledge test [13], and a six‐week social media intervention improved aspects of GF diet knowledge [14].

There are limitations in the study; our cohort had a predominantly white ethnicity, with 71% being female; thus, the results may not reflect the general coeliac population. In the future, with the development of generative AI technology, there is an opportunity to translate digital resources with little additional expenditure. The capturing of non‐participants’ demographic data allowed for the identification of selection bias. Most participants and non‐participants were female; coeliac disease is a female‐predominant condition. Participants and non‐participants were similar in ethnicity and age. There was a high attrition rate between pre‐ and post‐webinar surveys, which increases the probability of non‐response bias, whereby potentially the most satisfied patients were likely to complete the post‐webinar survey. The evaluation did not recruit a control group; it is possible that factors beyond the webinar were influencing the behaviours and knowledge acquisition; therefore, a future randomised controlled trial is advised.

## 5 Conclusion

The dietitian‐led pre‐recorded webinar improved patient outcomes and provided free access to digital health education for an unlimited number of patients, enabling timely self‐management post coeliac disease diagnosis. It could offer a solution to the poor access to specialist dietetic services. The webinar service enables specialist dietitians to utilise their skills and finite time for appointments with patients with lower health or digital literacy, minority ethnic groups where a translator is required and those with more complex health conditions.

## Author Contributions

**Leah Seamark:** conceptualization, data curation, writing – original draft (equal), project administration (lead), writing – review and editing (equal), **Olivia Radcliffe:** data curation, writing – review and editing (equal), **Marieanne Williams:** conceptualization, writing – review and editing (equal), **Jim Gotto:** writing – review and editing (equal), **Bridie Watson:** data curation, writing – review and editing (equal), **Jenna Trudgeon:** writing – review and editing (equal), **Sue Reeves:** writing – review and editing (equal), **Yvonne Jeanes:** visualization (equal), formal analysis, writing ‐ original draft preparation (equal).

## Funding

The authors have nothing to report.

## Conflicts of Interest

The authors declare no conflicts of interest.

## Supporting information


Supporting File

## Data Availability

The data that support the findings of this study are available on request from the corresponding author. The data are not publicly available due to privacy or ethical restrictions.
