# Supplementary material for: An Evaluation of a Dietitian‐Led Webinar for Adults With Coeliac Disease: A Positive Change in Gluten‐Free Food Knowledge and Behaviours
Source: J Hum Nutr Diet. 2026 Sep 23;39(5):e70350. doi: 10.1111/jhn.70350 (PMC13601820; doi:10.1111/jhn.70350)
Supplement: Supplementary file 1 [file JHN-39-0-s001.docx]

Supplementary file

Gluten Free foods knowledge score: Patients were asked if the following foods were allowed, a food to question or not allowed.

Cocoa

potato crisps

Croutons

Flavoured yoghurt

Meat sausages

fish fingers

Balsamic vinegar

Chickpea flour

Rice crisp cereal

Codex wheat starch

Soy sauce

Milk

Buckwheat

Modified maize starch

Spelt

Egg noodles

Porridge oats

Table 1.

|  | Did not watch/ partially watched*  N=19 | Accessed webinar non-participant  N=440 | Accessed webinar Pre survey only  N=302 | Accessed webinar Pre and post survey  N=100 |
| --- | --- | --- | --- | --- |
| Male/ female/ prefer not to say (%) | 32/68/0 | 30/ 66/ 3 | 27/ 72/ 1 | 26/ 71/ 1 |
| White ethnicity (%) | 100 | 87 | 94 | 95 |
| Age in years (mean ± SD) | 57.6 ± 19.9 | 48.0 ± 18.5 | 47.8 ±16.7 | 52.4±15.5 yrs |

*data from the paper survey.
